# A rare non-gadolinium enhancing sarcoma brain metastasis with microenvironment dominated by tumor-associated macrophages

**DOI:** 10.1186/s40478-023-01713-8

**Published:** 2024-01-22

**Authors:** David Rogawski, Joshua Wheeler, Esther Nie, William Zhu, Eleanor Villanueva, Gwen Coffey, Qian Ma, Kristen Ganjoo, Nancy Fischbein, Michael Iv, Hannes Vogel, Seema Nagpal

**Affiliations:** 1 Division of Neuro-Oncology, Stanford Medicine, Stanford, CA 94305 USA; 2 Division of Neuropathology, Department of Pathology, Stanford Medicine, Stanford, CA 94305 USA; 3 Division of Neuroimmunology, Stanford Medicine, Stanford, CA 94305 USA; 4 Department of Neurology and Neurological Sciences, Stanford Medicine, Stanford, CA 94305 USA; 5 Division of Oncology, Department of Medicine, Stanford Medicine, Stanford, CA 94305 USA; 6 Division of Neuroradiology, Department of Radiology, Stanford Medicine, Stanford, CA 94305 USA

**Keywords:** Sarcoma, Brain metastasis, Tumor-associated macrophages, Brain tumor microenvironment

## Abstract

Brain metastases occur in 1% of sarcoma cases and are associated with a median overall survival of 6 months. We report a rare case of a brain metastasis with unique radiologic and histopathologic features in a patient with low grade fibromyxoid sarcoma (LGFMS) previously treated with immune checkpoint inhibitor (ICI) therapy. The lone metastasis progressed in the midbrain tegmentum over 15 months as a non-enhancing, T2-hyperintense lesion with peripheral diffusion restriction, mimicking a demyelinating lesion. Histopathology of the lesion at autopsy revealed a rich infiltrate of tumor-associated macrophages (TAMs) with highest density at the leading edge of the metastasis, whereas there was a paucity of lymphocytes, suggestive of an immunologically cold environment. Given the important immunosuppressive and tumor-promoting functions of TAMs in gliomas and carcinoma/melanoma brain metastases, this unusual case provides an interesting example of a dense TAM infiltrate in a much rarer sarcoma brain metastasis.

## Introduction

The development of brain metastases depends on supportive interactions between cancer cells and the surrounding brain tumor microenvironment (TME). Neurons, glia, and immune cells support cancer growth through diverse mechanisms including neuron-to-cancer cell synapses, astrocyte-to-cancer cell gap junctions, and maintenance of immunosuppression [2, 8, 16, 18]. TAMs, which include CNS-resident microglia and monocyte-derived macrophages (MDMs) recruited from the periphery, are a major component of the TME with important tumor-promoting and immunosuppressive functions. Whereas the glioma TME contains abundant microglia, the TME of carcinoma and melanoma brain metastases contains a greater proportion of MDMs as well as infiltrating lymphocytes and neutrophils [4, 9]. Sarcoma brain metastases are very rare, occurring in 1% of cases [5], and the TME of these brain metastases has not been well studied. We present a case of a sarcoma brain metastasis with unique imaging and histopathologic features, including high density of TAMs in the TME.

## Case presentation

A 44-year-old woman with history of recurrent, metastatic LGFMS of the left piriformis muscle previously treated with ICIs presented with 1 month of gradually worsening binocular diagonal diplopia. Over the ten years prior to onset of neurologic symptoms, the LGFMS was treated with three surgical resections in the left gluteal region (pathology showed Fédération Nationale des Centres de Lutte Contre le Cancer grade 2 of 3, all resections had positive margins), two courses of radiation to the left pelvic region, and resection of a right cervical paraspinal metastasis followed by adjuvant radiation. Two years prior to presentation, there was progression of sarcoma in the pelvis as well as metastatic disease in the lungs, kidneys, and bone, with pathologic confirmation of bone involvement. She was started on a clinical trial in which she received IV ipilimumab 1 mg/kg and IV nivolumab 3 mg/kg (ICIs) every 3 weeks for four cycles, followed by nivolumab 480 mg every 4 weeks. Cryoablation of a left gluteal mass was performed after the first infusion of ipilimumab and nivolumab. ICI treatment was complicated by thyroiditis, adrenal insufficiency, and hepatitis that were managed with corticosteroids and hormone replacement. ICIs were discontinued after 9 months of therapy, 8 months prior to her presentation with neurologic symptoms. At the time of diplopia onset, there was a growing left adrenal metastasis being monitored on PET/CT. Neurologic exam showed right hypertropia worse with right and down gaze, and mild bilateral ptosis.

**Fig. 1:**
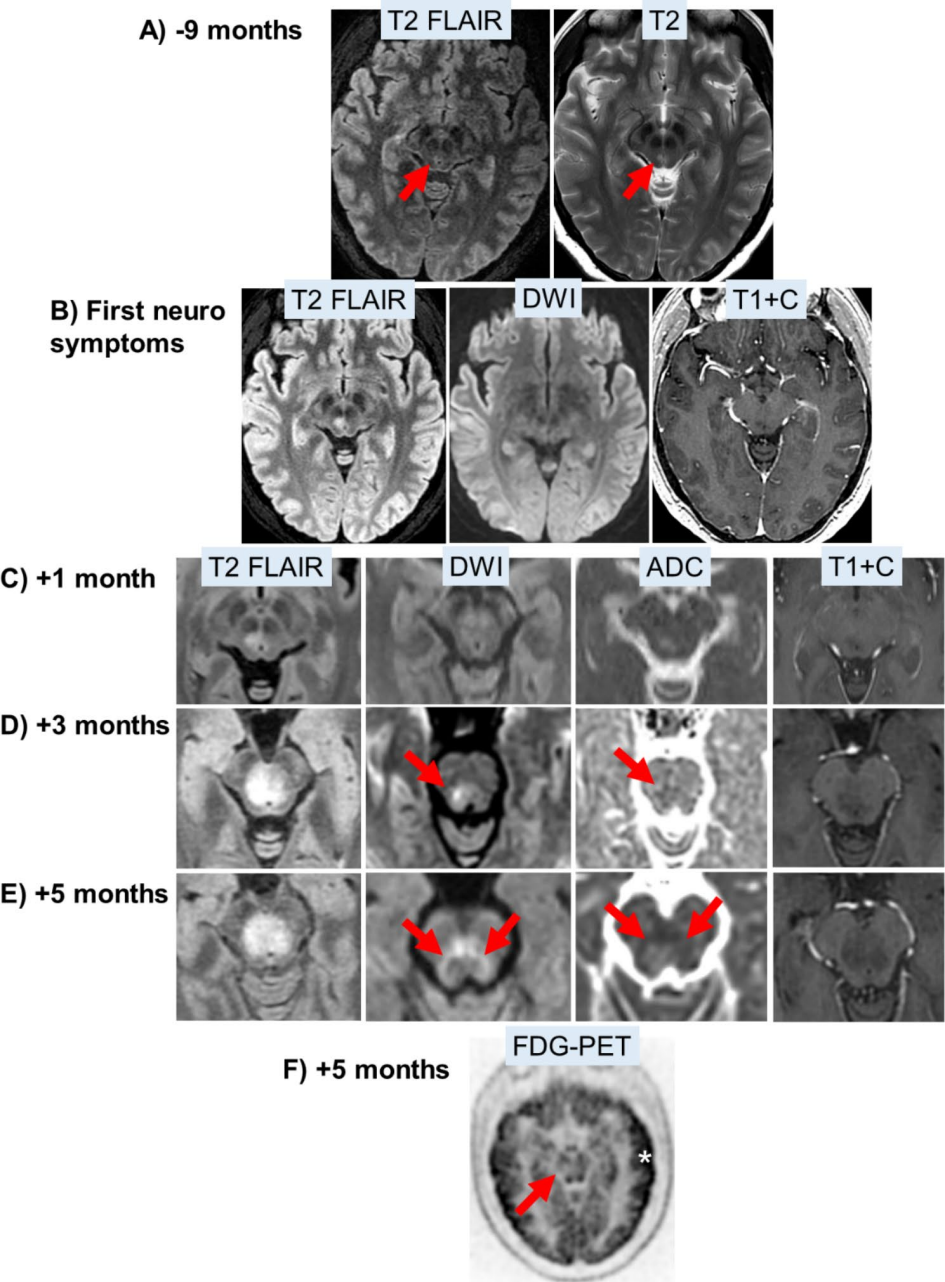
Radiologic progression of midbrain lesion. (A) Subtle T2/FLAIR-hyperintense lesion (red arrow) seen in retrospect 9 months prior to presentation with neurologic symptoms. (B) Right paramedian midbrain lesion at onset of neurologic symptoms, without contrast enhancement or diffusion restriction. (C-E) Growth of the lesion over 5 months, with gradual development of peripheral diffusion restriction (red arrows) but still no contrast enhancement. (F) FDG-PET shows midbrain lesion (red arrow) is hypometabolic compared to grey matter (*).

MRI of the brain revealed an 8 mm T2 hyperintense lesion in the right paramedian midbrain without restricted diffusion or enhancement. In retrospect, the midbrain lesion was subtly present on an MRI obtained 9 months prior (Fig. 1A, B). An extensive infectious and inflammatory workup including serum MOG-IgG and AQP-4 IgG antibodies and cerebrospinal fluid analysis including cytology, flow cytometry, oligoclonal bands, JC virus PCR, and autoimmune antibody panel was negative. MRI of the spine was negative for spinal cord lesions. The suspected diagnosis was CNS demyelination secondary to ICI, and she was treated with IV methylprednisolone 1 g for three days, followed by maintenance oral prednisone (30–60 mg daily), without improvement in her diplopia.

Three months after her presentation with diplopia, she developed fatigue accompanied by severe bilateral ptosis and ophthalmoplegia and left arm ataxia. MRI showed expansion of the non-enhancing T2-hyperintense midbrain lesion, now extending into the right medial thalamus and pons (Fig. 1C, D). The differential diagnosis was expanded to include glioma, however biopsy was deemed unsafe given the rostral brainstem location. Metastasis was not considered given the infiltrative and non-enhancing nature of the lesion. Further empiric treatment for an autoimmune etiology was given with IVIg 2 g/kg over three days. The patient underwent resection of the left adrenal mass, with pathology demonstrating metastatic sarcoma with high grade features including elevated mitotic rate of at least 30 mitotic figures per 10 high-power fields and 20% of the tumor with necrosis. The morphologic and immunophenotypic findings from the adrenal resection were interpreted as most consistent with sclerosing epithelioid fibrosarcoma (SEF), and the classic features of LGFMS were not seen. The high grade features consistent with SEF in the adrenal mass likely represented progression of the patient’s prior LGFMS.

Four months after presentation she developed worsening balance and falls and underwent five sessions of plasma exchange over six days. Five months after presentation she developed left-sided weakness, dysarthria and somnolence suggestive of involvement of the reticular activating system. MRI showed continued mild increase in the size of the midbrain lesion, with gradual development of peripheral diffusion restriction but still no contrast enhancement (Fig. 1E). PET/CT showed the midbrain lesion to be hypometabolic compared to grey matter (Fig. 1F), favoring an inflammatory rather than neoplastic process. She developed dysphagia requiring PEG tube placement and urinary retention requiring intermittent catheterization. Her clinical status deteriorated further over the next month with non-convulsive status epilepticus treated with levetiracetam and lacosamide. Despite resolution of seizures, her mental status remained depressed requiring invasive ventilation. She was transitioned to hospice care and died 6 months after presenting with neurologic symptoms.

**Fig. 2:**
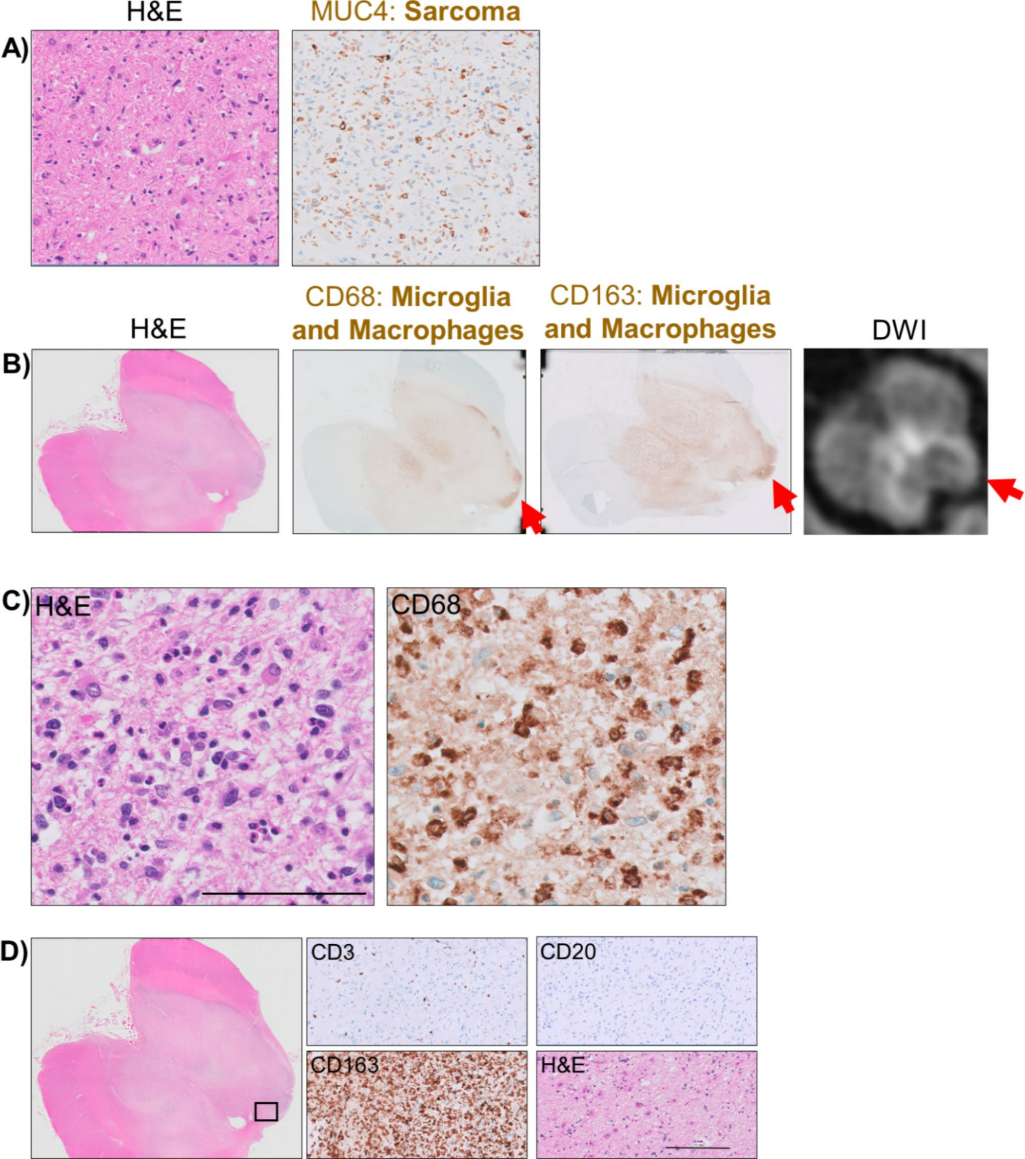
Histopathologic findings in the midbrain at autopsy. (A) Lesion contains MUC4 + sarcoma cells. (B) Extensive infiltration of CD68 + and CD163 + TAMs, with highest density at the leading edge of the metastasis corresponding to diffusion restriction on MRI (red arrows). (C) High magnification images of lesion depict morphology of CD68 + TAMs. Scale bar = 100 μm. (D) Immunohistochemical staining within the boxed region at left reveals a paucity of CD3 + T cells and CD20 + B cells compared to abundant CD163 + TAMs. Scale bar = 250 μm

Autopsy revealed metastatic sarcoma involving the midbrain, pons, and thalami (Fig. 2A). The sarcoma centered on the periaqueductal region with accompanying disseminated single-cell infiltration throughout the midbrain. The neoplastic cells stained positive for MUC-4 (Fig. 2A) and harbored a *TAOK1::FUS* rearrangement. While *FUS* fusions are common in some sarcoma subtypes, the *TAOK1::FUS* fusion has not been previously reported. The lesional midbrain and periaqueductal parenchyma were notable for extensive infiltration of CD68 + and CD163 + TAMs (Fig. 2B, C). The number of TAMs was particularly extensive relative to the number of tumor cells present, with highest TAM density at the leading edge of the metastasis, a location correlating with diffusion restriction on MRI (Fig. 2B, rightmost panel). In contrast to the abundant TAMs, there was a relative paucity of lymphocytes including CD3 + T cells and CD20 + B cells (Fig. 2D). Lymphocyte counts across 10 randomly sampled 1 mm^2^ regions revealed 17 ± 10 (mean ± standard deviation) CD3 + T cells per mm^2^ and 2 ± 1 CD20 + B cells per mm^2^. There was no evidence of tumor infiltrating the leptomeninges.

## Discussion

LGFMS has an incidence of 0.18 per million and typically presents in the extremities and trunk [13]. LGFMS may metastasize to the lungs and retroperitoneum [13], but there are no previous reports of brain metastases from systemic sites. There are six reports of primary intracranial LGFMS, all of which were well-circumscribed enhancing masses [1, 20]. SEF is a rare and aggressive soft-tissue sarcoma first described as a variant of LGFMS. LGFMS and SEF may appear together as a hybrid tumor, and they share diagnostic features including MUC4 immunoreactivity and *FUS* gene rearrangements [19]. SEF has distant metastatic spread in 40–80% of cases, and in a series of 88 patients with metastatic SEF, 2.9% had brain metastases [21]. This case represents an already rare sarcoma brain metastasis with additional unique and confounding radiologic and histopathologic features.

The sarcoma brain metastasis microenvironment featured extensive TAM infiltration and a dearth of lymphocytes. This contrasts with the relative abundance of lymphocytes found in the TME of more common brain metastases from breast, lung, and melanoma tumors [4, 9]. Interestingly, this case shared some characteristics with diffuse midline glioma (DMG), including the lack of enhancement and brainstem location, and DMG also has a non-inflammatory immune environment with abundance of TAMs, low levels of inflammatory cytokines, and a paucity of lymphocytes [10]. TAMs promote tumor growth by inducing immunosuppression, remodeling the extracellular matrix, and stimulating angiogenesis [16, 17, 22]. In vitro models of brain metastasis show microglia attaching themselves to tumor cells and actively transporting them into brain tissue [15]. TAMs have an inhibitory effect on T cell recruitment and function [3], which may have contributed to the paucity of lymphocytes and weak immune response in this case. Even if this patient had been eligible for further ICIs, the lack of lymphocytes suggests the lesion would have responded poorly to immunotherapy [11].

Peripheral diffusion restriction is rare for brain metastases and is more commonly seen in central nervous system demyelinating lesions, where it is attributed to clustering of activated microglia [6]. The peripheral diffusion restriction in this case coincided with dense infiltration of TAMs at the leading edge of the brain metastasis, implying that tightly packed TAMs restricted water diffusion akin to demyelinating lesions. Additionally, the persistent lack of contrast enhancement is unusual for brain metastases, which are typically fueled by vascular endothelial growth factor (VEGF)-dependent angiogenesis associated with a disrupted blood-brain barrier. Non-enhancement of brain metastases may occur after receipt of the anti-VEGF antibody bevacizumab [7], which this patient did not receive. Thus, the absence of contrast enhancement in this case suggests that VEGF-mediated angiogenesis was not required for metastasis growth. Interestingly, TAMs mediate resistance to antiangiogenic brain tumor therapies in preclinical models, and TAMs are increased at autopsy in recurrent glioblastoma patients who received antiangiogenic therapies compared with patients who did not [12, 14]. Rebound vascularization does not occur in TAM-mediated antiangiogenic resistance, underscoring that TAMs support cancer cells in diverse ways such as promoting tumor cell migration, invasion, and immunosuppression [12]. This case with extensive TAMs and an intact blood-brain barrier is consistent with prior studies showing that TAMs facilitate brain tumor growth in many ways beyond angiogenesis.

In sum, we report an extremely rare fibromyxoid sarcoma brain metastasis with unique radiologic and histopathologic features, highlighting the role of the TME and showing that non-enhancing lesions may represent brain metastases in certain contexts. The dense infiltration of TAMs together with scarce lymphocytes seen in this case raises the question whether TAMs might have important immunosuppressive and tumor-promoting roles in sarcoma brain metastases, similar to other more common brain tumors. Future research will determine if therapies that alter TAM phenotypes to create a hostile TME are effective as brain tumor treatments.

## Data Availability

All data generated or analyzed during this study are included in this published article.

## Abbreviations

TAM: Tumor-associated macrophages
ICI: Immune checkpoint inhibitor
TME: Tumor microenvironment
VEGF: Vascular endothelial growth factor
LGFMS: Low grade fibromyxoid sarcoma
MDM: Monocyte-derived macrophages
SEF: Sclerosing epithelioid fibrosarcoma
TIL: Tumor-infiltrating lymphocyte
